# Immune checkpoint inhibitor plus tyrosine kinase inhibitor with or without transarterial chemoembolization for unresectable hepatocellular carcinoma

**DOI:** 10.3389/fonc.2025.1385304

**Published:** 2025-03-10

**Authors:** Hongyu Pan, Minghao Ruan, Riming Jin, Jin Zhang, Yao Li, Dong Wu, Lijie Zhang, Wen Sun, Ruoyu Wang

**Affiliations:** ^1^ The First Department of Hepatic Surgery, Eastern Hepatobiliary Surgery Hospital, The Naval Medical University, Shanghai, China; ^2^ The Department of Information, Changhai Hospital, Naval Medical University, Shanghai, China; ^3^ National Center for Liver Cancer, The Naval Medical University, Shanghai, China

**Keywords:** hepatocellular carcinoma, immune checkpoint inhibitors, tyrosine kinase inhibitors, transcatheter arterial chemoembolization, combination therapy

## Abstract

**Background and aims:**

Transcatheter arterial chemoembolization (TACE) has been combined with immune checkpoint inhibitor (ICI)-based systemic therapies for unresectable hepatocellular carcinoma (uHCC) with promising efficacy. However, whether the addition of TACE to the combination of ICI and tyrosine kinase inhibitor (TKI) (ICI+TKI+TACE) is superior to ICI+TKI combination therapy is still not clear. Thus, this study compares the efficacy of ICI+TKI+TACE triple therapy and ICI+TKI doublet therapy in patients with uHCC.

**Methods:**

uHCC patients treated with either ICI+TKI+TACE triple therapy or ICI+TKI doublet therapy were retrospectively recruited between January 2016 and December 2021 at Eastern Hepatobiliary Surgery Hospital. The patients from ICI+TKI+TACE group and ICI+TKI group were further subjected to propensity score matching (PSM). The primary outcome was progression-free survival (PFS). The secondary outcomes were overall survival (OS) and objective response rate (ORR). Post-progression survival (PPS) as well as treatment-related adverse events (TRAEs) were also assessed.

**Results:**

A total of 120 patients were matched. The median PFS was 8.4 months in ICI+TKI+TACE triple therapy group versus 6.6 months in ICI+TKI doublet therapy group (HR 0.72, 95%CI 0.48-1.08; *p*=0.115). Similar results were obtained in term of OS (26.9 versus 24.2 months, HR 0.88, 95% CI 0.51-1.52; *p*=0.670). The ORR in the triple therapy group was comparable with that in the doublet therapy group (16.6% versus 21.6%, *p*=0.487). Further subgroup analysis for PFS illustrated that patients without previous locoregional treatment (preLRT) (10.5 versus 3.7 months, HR 0.35 [0.16-0.76]; *p*=0.009), without previous treatment (10.5 versus 3.5 months, HR 0.34 [0.14-0.81]; *p*=0.015) or treated with lenvatinib (14.8 versus 6.9 months, HR 0.52 [0.31-0.87]; *p*=0.013) can significantly benefit from triple therapy compared with doublet therapy. A remarkable interaction between treatment and preLRT (*p*=0.049) or TKIs-combined (*p*=0.005) was also detected in term of PFS. Post progression treatment significantly improved PPS in both groups. The incidence of TRAEs was comparable between two groups.

**Conclusions:**

The addition of TACE to ICI+TKI combination therapy did not result in a substantial improvement in efficacy and prognosis of patients. However, in selected uHCC patients (without preLRT or treated with lenvatinib as combination), ICI+TKI+TACE triple therapy may remarkably improve PFS.

## Introduction

Hepatocellular carcinoma (HCC) is the sixth most common cancer globally and the third leading cause of cancer-related mortality (1). Surgical resection is potentially curative for patients with early-stage HCC, but about 50-70% of HCC patients are unfit for surgical resection due to advanced tumor stage (2). For patients with advanced HCC, multitarget tyrosine kinase inhibitors (TKIs) sorafenib and lenvatinib used to be the first line treatment, but the efficacy was modest and only conferred limited survival benefits. Recently, immune checkpoint inhibitors (ICIs) including anti-programmed death-1 (PD-1)/programmed death-ligand 1 (PD-L1) monoclonal antibodies have shown encouraging results in multiple cancers (3, 4). The IMbrave150 trial demonstrated a better tumor response and survival with the combination of atezolizumab and bevacizumab versus sorafenib (5), resulting in the accelerated approval of this combination for advanced HCC, which also started a new tide of clinical studies leading by ICI-based combination therapy in HCC. Among these, the combination of TKI and ICI was also investigated and promising efficacy was reported in the KEYNOTE 524 trial with a median progression‐free survival (PFS) of 9.3 months and a median overall survival (OS) of 22 months for patients treated with pembrolizumab plus lenvatinib (6). But subsequent phase III study LEAP-002 which compared pembrolizumab plus lenvatinib with lenvatinib failed to meet its primary endpoint (7). However, TKIs and their combination with ICIs are still important treatment options for advanced HCC.

On the other hand, although atezolizumab plus bevacizumab is now the preferred first-line therapy for advanced HCC, more than 50% of patients still do not respond to treatment. To further increase the objective response rate (ORR) and improve patient survival, loco-regional treatments (LRTs) including transcatheter arterial chemoembolization (TACE), radiotherapy (RT) and hepatic arterial infusion chemotherapy (HAIC) have been combined with ICI-based systemic therapies for unresectable HCC (uHCC) (8, 9). TACE, which is the first therapeutic modality to provide survival benefits for patients with uHCC (10), is the standard treatment for Barcelona Clinic Liver Cancer (BCLC) stage B HCC in western countries (11) and BCLC stage B/C HCC in Asia‐Pacific (12). The results of LAUNCH and TACTICS trial showed that TACE combined with TKI significantly prolonged the survival of HCC patients (13, 14). Mechanistically, the upregulated expression of vascular endothelial growth factor (VEGF) and fibroblast growth factor (FGF) by TACE (15) could be effectively inhibited by TKIs (16), leading to better clinical outcomes in patients treated with TACE plus TKIs (17). In addition, the potential benefit of TACE plus PD‐1 inhibitor has also been revealed. A retrospective study has reported that TACE can be safely integrated with PD-1 inhibitor and lead to significant delay in tumor progression and disease downstaging in selected patients (18). Meanwhile, TACE with or without lenvatinib plus pembrolizumab is under investigation for intermediate-stage HCC not amenable to curative treatment in phase 3 LEAP-012 study (19). However, currently, whether the addition of TACE to the combination of TKIs and PD-1 inhibitor (ICI+TKI+TACE) is superior to ICI+TKI combination therapy is still not clear. This study leverages a retrospective cohort of HCC patients treated with ICI+TKI+TACE triple therapy or ICI+TKI doublet therapy to examine the differences of efficacy and patient outcome between the two regimens.

## Methods

### Study design and patients

This retrospective study was conducted on adult patients diagnosed with uHCC at Eastern Hepatobiliary Surgery Hospital, Shanghai, China, from January 2016 to December 2021. This study was approved by the Institutional Ethics Committee of Eastern Hepatobiliary Surgery Hospital and conducted in strict accordance with the principles of the Declaration of Helsinki. As patient identities were anonymized, the requirement for informed consent was waived by the Ethics Committees.

**Figure 1:**
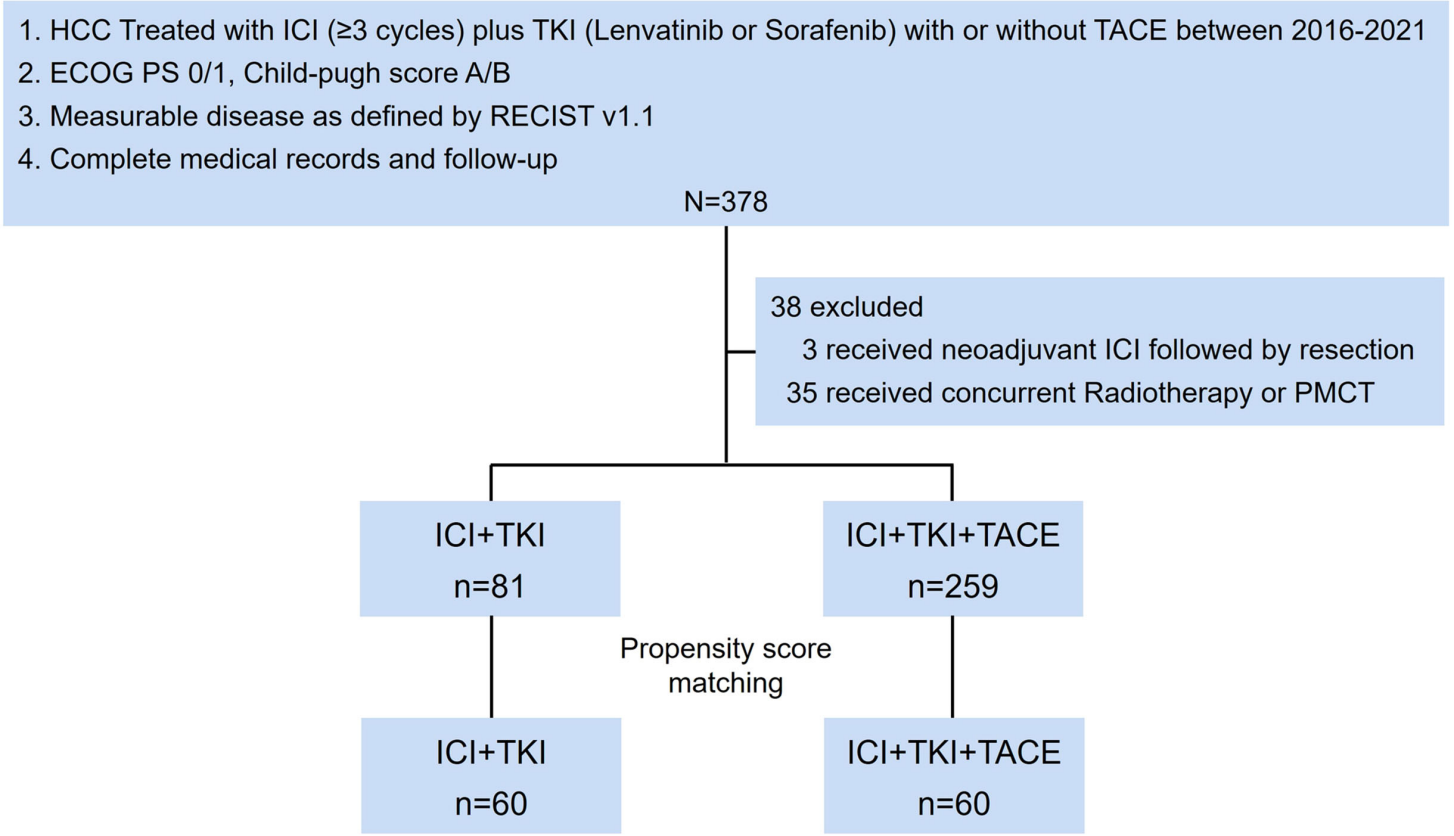
Study flowchart. TACE, transarterial chemoembolization; TKIs, Tyrosine kinase inhibitors; ICI, immune checkpoint inhibitor; PMCT, percutaneous microwave coagulation therapy.

The main inclusion criteria of patients were as follows: (1) Patients with unresectable or metastatic, histologically, or radiographically diagnosed HCC. Patients were classified as unresectable if R0 resection is impossible, or remnant liver volume is below 30% in non-cirrhotic patients or 40% in cirrhotic patients, or tumor stage is BCLC stage B and up-to-seven criteria out (20), or stage C; (2) Eastern Cooperative Oncology Group performance status (ECOG PS) 0 or 1; (3) preserved liver function (Child-Pugh A or B); (4) measurable disease as defined by Response Evaluation Criteria in Solid Tumors version 1.1 (RECIST v1.1); (5) complete medical records and follow-up; (6) patients were treated with ICI (anti-PD-1/PD-L1 monoclonal antibody, ≥3 cycles) plus TKIs (lenvatinib or sorafenib) (ICI+TKI) with or without TACE (**Figure 1**). TACE was performed within 30 days before or after the start of ICI+TKI, with 15 patients received simultaneous TACE and systemic therapy, 37 patients initiated TACE before systemic therapy (median interval: -5 days, range: -1 to -23 days; 95% CI: -22, -2) and 8 patients started TACE after systemic therapy (median interval: +7.5 days, range: +1 to +16 days; 95% CI: +1, +16). The overall median interval from TACE to systemic therapy was +3 days (TACE was performed 3 days post systemic therapy) (range: -23 to +16 days, 95% CI: -18.5, +9 days). A total of 378 patients were screened for eligibility, and 38 patients were excluded (**Figure 1** and **Supplementary Table S1**). Of the 340 patients included, 259 patients received ICI+TKI+TACE triple treatment and 81 received ICI+TKI doublet treatment. Their detailed clinicopathologic features are described in **Supplementary Table S1**. Propensity score (PS) matching was further performed to match patients from triple treatment and doublet treatment group. The diagram with the flow of patients was shown in **Figure 1**.

### TACE procedure

The vascular catheter was inserted through a femoral artery using the Seldinger technique to the hepatic artery, then tumor-feeding arteries were identified by angiography, and lipiodol emulsion mixed with doxorubicin hydrochloride or pirarubicin was administered into the tumor-feeding vessels. Subsequently, embolization was performed with the injection of polyvinyl alcohol particles and absorbable gelatin sponge particles until complete arterial flow stasis was observed. The amounts of anticancer agent and lipiodol were adjusted according to the body surface area of the patient and liver function. According to physicians’ assessment, TACE was conducted repeatedly on demand, mainly based on the proportion of active area tumors and status of hepatic function.

### Objectives and assessments

The primary outcome was progression-free survival (PFS). The PFS was defined as the time interval between the initiation of TACE or systemic therapy, whichever comes first, and disease progression or death from any cause. The secondary outcomes included overall survival (OS) and objective response rate (ORR). The OS was defined as the time from the initiation of TACE or systemic therapy whichever comes first to death from any cause. The ORR was defined as the proportion of patients with a confirmed complete/partial response (CR/PR) as best overall response (BOR) according to RECIST v1.1. Post progression treatments were also recorded. PPS was defined as the time from first progression upon ICI+TKI or ICI+TKI+TACE therapy to death from any cause. Treatment-related adverse events were extracted and reviewed by two physicians from the medical records.

### Statistical analysis

All clinical data were analyzed using IBM SPSS Statistics 26 software. Student’s *t* test was used to compare continuous variables, and the χ2 test or Fisher’s exact test was used to compare categorical variables. The PSM method was applied to balance the patients from triple treatment and doublet treatment group (**Supplementary Table S1**) using SPSS software PS Matching procedure. The balancing caliper was set at 0.2. Balancing covariates included age, gender, HBV, HCV, ECOG PS, Child-pugh score, previous loco-regional treatment (preLRT), previous TKI treatment (preTKI), First-line treatment, previously untreated, macrovascular invasion (MVI), extrahepatic metastasis, BCLC stage, TKIs-combined, DCP and AFP levels at baseline. After PS matching, 60 of 259 patients treated with ICI+TKI+TACE triple therapy were matched to 60 patients who had received ICI+TKI doublet therapy by PSs (**Table 1**). Survival curves were calculated using the Kaplan-Meier method and compared using the log‐rank test. Univariate and multivariate Cox regression analysis was used to identify potential risk factors associated with survival. All *p* values were 2 sided, and *p*<0.05 was considered statistically significant.

**Table 1:** Baseline characteristics of patients matched by PSs.

	No.(%)		
Characteristic	ICI+TKI+TACE group (n=60)	ICI+TKI group (n=60)	*p* value
Age(years)			
≥60	42 (70.00%)	47 (78.33%)	0.297
<60	18 (30.00%)	13 (21.67%)	
Gender			
Male	49 (81.67%)	52 (86.67%)	0.453
Female	11 (18.33%)	8 (13.33%)	
HBV			
Positive	55 (91.67%)	57 (95.00%)	0.464
Negative	5 (8.33%)	3 (5.00%)	
HCV			
Positive	0 (0.00%)	1 (1.67%)	0.315
Negative	60 (100.00%)	59 (98.33%)	
EGOG PS			
1	31 (51.67%)	30 (50.00%)	0.855
0	29 (48.33%)	30 (50.00%)	
Child-Pugh Score			
A	52 (86.67%)	52 (86.67%)	1.000
B	8 (13.33%)	8 (13.33%)	
preLRT			
Yes	41 (68.33%)	41 (68.33%)	1.000
No	19 (31.67%)	19 (31.67%)	
preTKI			
Yes	11 (18.33%)	15 (25.00%)	0.375
No	49 (81.67%)	45 (75.00%)	
First-line			
Yes	49 (81.67%)	45 (75.00%)	0.375
No	11 (18.33%)	15 (25.00%)	
Previously Untreated			
Yes	15 (25.00%)	16 (26.67%)	0.835
No	45 (75.00%)	44 (73.33%)	
MVI			
present	19 (31.67%)	23 (38.33%)	0.444
absent	41 (68.33%)	37 (61.67%)	
Extrahepatic Metastasis			
present	26 (43.33%)	30 (50.00%)	0.464
absent	34 (56.67%)	30 (50.00%)	
BCLC stage			
A	9 (15.00%)	7 (11.67%)	0.221
B	18 (30.00%)	11 (18.33%)	
C	33 (55.00%)	42 (70.00%)	
TKIs-combined			
Sorafenib	18 (30.00%)	17 (28.33%)	0.841
Lenvatinb	42 (70.00%)	43 (71.67%)	
DCP(mAU/mL)			
≥400	32 (53.33%)	35 (58.33%)	0.581
<400	28 (46.67%)	25 (41.67%)	
AFP(μg/L)			
≥400	20 (33.33%)	20 (33.33%)	1.000
<400	40 (66.67%)	40 (66.67%)	

HBV, hepatitis B virus; HCV, hepatitis C virus; TKI, Tyrosine kinase inhibitor; ECOG PS, Eastern Cooperative Oncology Group Performance Status; preLRT, Previous loco-regional therapy; MVI, Macrovascular Invasion; BCLC stage, Barcelona Clinic Liver Cancer stage; AFP, Alpha-fetoprotein; DCP, Des-gamma-carboxy prothrombin.

## Results

### Patients and treatment

Between January 2016 and December 2021, 378 patients with HCC at the Eastern Hepatobiliary Surgery Hospital were screened for eligibility, and 38 patients were excluded (**Figure 1** and **Supplementary Table S1**). A total of 81 patients have received ICI+TKI doublet therapy and 259 patients have received ICI+TKI+TACE triple therapy. The baseline characteristics of included patients were listed in **Supplementary Table S1**. The included patients from ICI+TKI group and ICI+TKI+TACE group were further subjected to PS matching based on the baseline characteristics. Sixty patients from ICI+TKI+TACE group were matched to 60 patients from ICI+TKI group and the baseline characteristics were well balanced between the two groups (**Table 1**). As of December 30, 2021, the median duration of follow-up was 23.9 months (24.2 months for ICI+TKI+TACE group, 23.9 months for ICI+TKI group).

### Survival analysis

**Figure 2:**
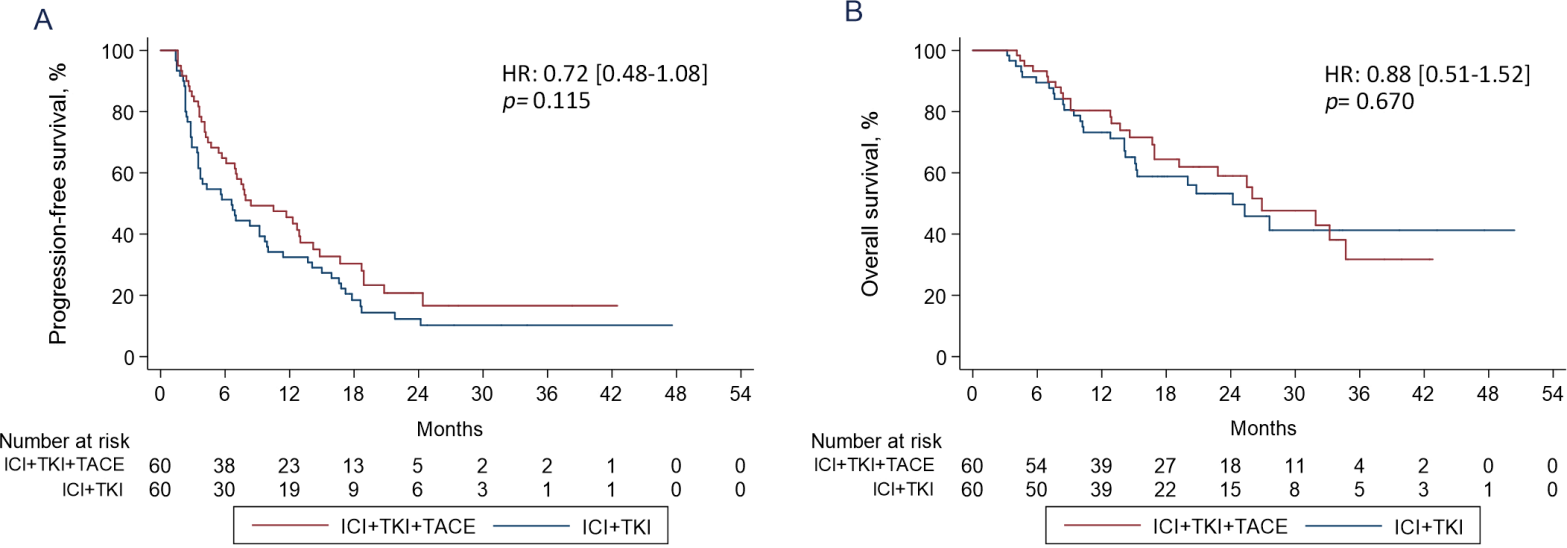
Kaplan-Meier estimates of PFS **(A)** and OS **(B)** curves in HCC patients treated with ICI+TKI+TACE or ICI+TKI therapy.

After PSM, the median PFS of patients in ICI+TKI+TACE triple therapy group was 8.4 months (95% CI 6.1-13) versus 6.6 months (95% CI 3.5-9.7) in ICI+TKI doublet therapy group (HR 0.72 [0.48-1.08]; *p*=0.115) (**Figure 2A**). The median OS of patients in the triple therapy group was also comparable with that in the doublet therapy group (26.9 versus 24.2 months, HR 0.88 [0.51-1.52]; *p*=0.670); (**Figure 2B**). Prior to PSM, the PFS (HR 0.82, 95% CI 0.61-1.09, p=0.185) and OS (HR 0.89, 95% CI 0.65-1.22, p=0.453) were comparable between ICI+TKI+TACE triple therapy group and ICI+TKI doublet therapy group, which were consistent with the outcomes after PSM (**Supplementary Figure S1**). Univariate analyses revealed that Child-Pugh score (B versus A, HR 2.18 [1.21-3.91]; *p*=0.009), macroscopic vascular invasion (MVI) (present versus absent, HR 1.55 [1.01-2.35]; *p*=0.041), TKIs-combined (lenvatinib versus sorafenib, HR 0.47 [0.30-0.72]; *p*=0.001) and des-gamma‐carboxy prothrombin (DCP) levels (>400 versus ≤400 mAU/mL, HR 1.71 [1.13-2.57]; *p*=0.010) were significantly associated with PFS (**Supplementary Table S2**). Multivariate analysis showed that Child-Pugh score (B versus A, HR 1.84 [1.01-3.37]; *p*=0.046) and TKIs-combined (lenvatinib versus sorafenib, HR 0.53 [0.34-0.82]; *p*=0.005) were identified as independent prognostic factors for PFS (**Supplementary Table S2**). Univariate analysis showed that Child-Pugh score (B versus A, HR 3.68 [1.85-7.31]; *p*=0.000), MVI (present versus absent, HR 2.85 [1.64-4.94]; *p*=0.000) and DCP (>400 versus ≤400 mAU/mL, HR 2.12 [1.19-3.78]; *p*=0.011) were significantly associated with OS (**Supplementary Table S3**). Child-Pugh score (B versus A, HR 3.11 [1.56-6.22]; *p*=0.001), MVI (present versus absent, HR 2.54 [1.46-4.43]; *p*=0.001) and DCP (>400 versus ≤400 mAU/mL, HR 1.90 [1.06-3.40]; *p*=0.029) were also identified as independent prognostic factors for OS (**Supplementary Table S3**).

### Tumor response

The BOR was shown in **Supplementary Table S4**. Ten (16.6%) patients in ICI+TKI+TACE triple therapy group and 13 (21.6%) patients in ICI+TKI doublet therapy group achieved CR/PR. Twenty (33.3%) and 31 (51.6%) patients achieved SD (stable disease) in triple therapy group and doublet therapy group, respectively. The ORR in the triple therapy group was comparable with that in the doublet therapy group (21.6% versus16.6%, *p*=0.487).

### Subgroup analysis

**Figure 3:**
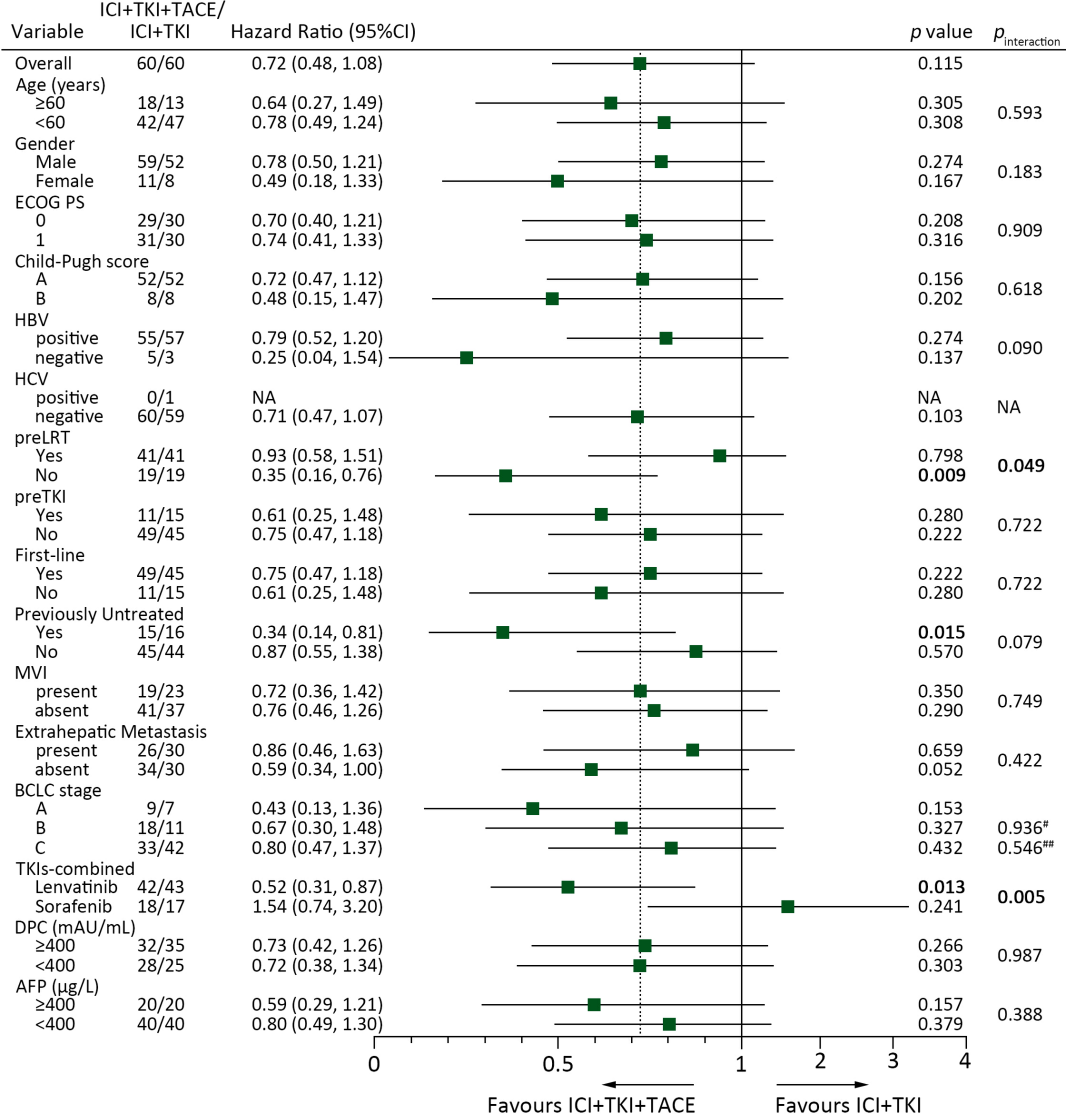
Subgroup COX proportional hazards regression model analysis of PFS according to the baseline characteristics and different treatment groups. ^#^, BCLC stage B versus A; ^##^, BCLC stage C versus A.

**Figure 4:**
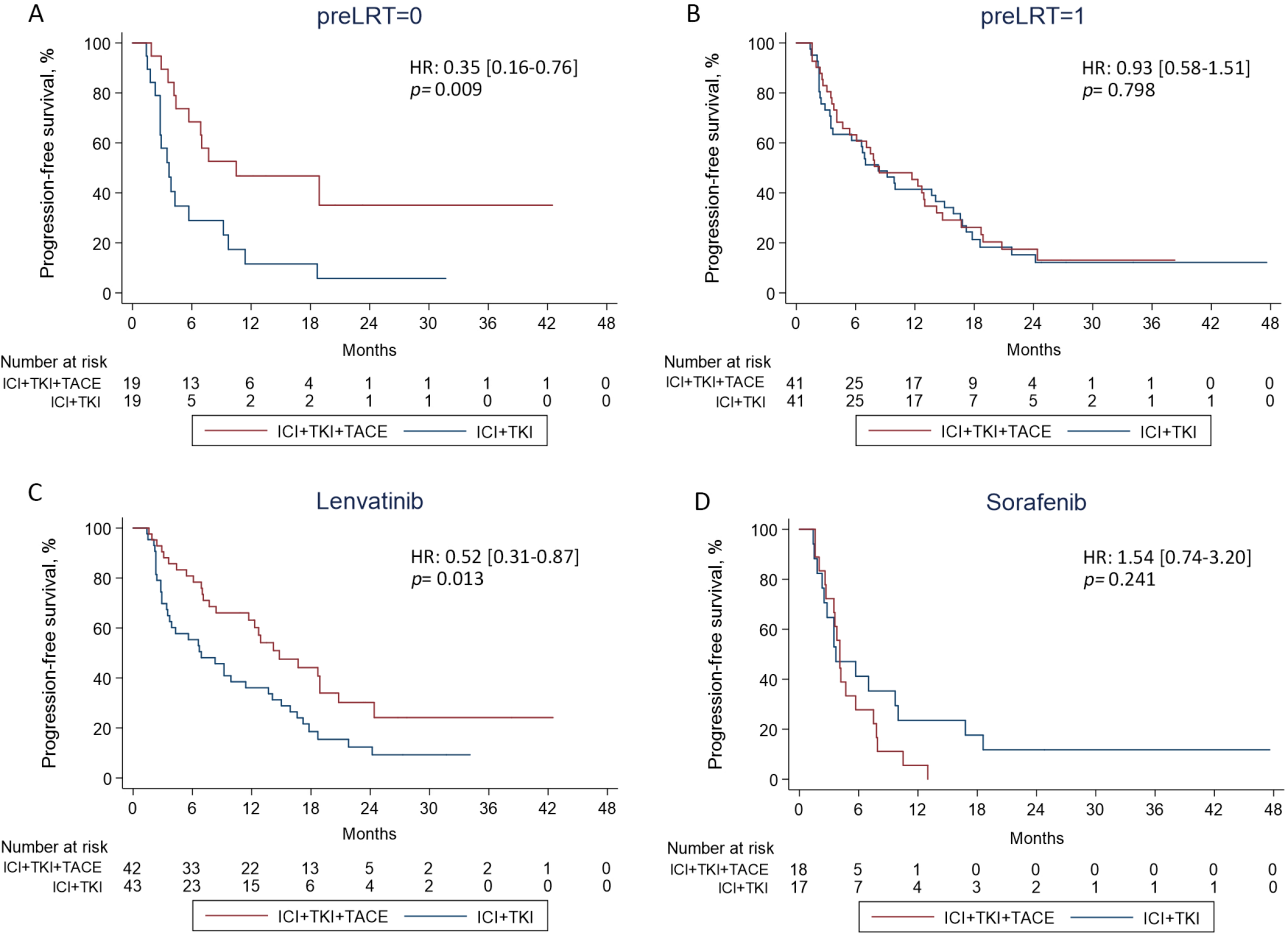
**(A, B)** The PFS was compared between the double and triple therapy groups in patients with or without preLRT. **(C, D)** The PFS was compared between the triple and doublet therapy groups in patients treated with lenvatinib or sorafenib.

To determine potential factors affecting patients’ response to ICI+TKI+TACE or ICI+TKI therapy, subgroup analysis was further performed. Subgroup analysis for PFS illustrated that patients with no preLRT (10.5 versus 3.7 months, HR 0.35 [0.16-0.76]; *p*=0.009), with no previous treatment (10.5 versus 3.5 months, HR 0.34 [0.14-0.81]; *p*=0.015) or treated with lenvatinib (14.8 versus 6.9 months, HR 0.52 [0.31-0.87]; *p*=0.013) can significantly benefit from triple therapy compared with doublet therapy (**Figure 3**). In addition, a remarkable interaction between treatment and preLRT (*p*=0.049) or TKIs-combined (*p*=0.005) was also detected (**Figure 3**). Kaplan-Meier analysis also observed that patients without preLRT or treated with lenvatinib displayed notable improved PFS upon ICI+TKI+TACE therapy versus ICI+TKI therapy (**Figures 4A, C**), while in patients with preLRT or treated with sorafenib, the PFS was comparable between ICI+TKI+TACE and ICI+TKI group (**Figures 4B, D**). Subgroup analysis for OS illustrated that only patients without preLRT favored triple therapy compared with doublet therapy (26.0 versus 15.1 months, HR 0.36 [0.13-0.96]; *p*=0.043) (**Supplementary Figure S2**). However, the interaction analysis didn’t detect significant interactions between treatment and patient baseline characteristics for OS. In addition, the efficacy of different therapeutic regimens across BCLC stages was also compared. The results showed that in patients with BCLC stage A/B, patients treated with ICI+lenvatinib+TACE exhibited significantly prolonged PFS compared to those treated with ICI+lenvatinib (*p*= 0.023), while their OS was not significantly improved (**Supplementary Figure S3**). Furthermore, in patients with BCLC stage C, no difference of PFS and OS was observed between triple therapy and doublet therapy in lenvatinib or sorafenib subgroup (**Supplementary Figure S3**). Moreover, a total of 12 patients have subsequently undergone conversion surgery, with 7 (40.0%) patients in the triple therapy group and 5 (45.0%) patients in the doublet therapy group (**Supplementary Table S5**). The number of patients underwent conversion surgery was comparable between the two groups (*p*=0.762). In addition, the PFS was significantly superior in patients who underwent conversion surgery compared to those who did not (HR 0.39 [0.19-0.90]; p=0.027). However, OS was not improved (HR 0.32 [0.07-1.33]; p=0.118). There were no significant differences in PFS or OS between patients who underwent conversion surgery in ICI+TKI+TACE group and ICI+TKI group (**Supplementary Figure S4**).

### Subsequent therapy post progression

Seventy-eight point eight (41/52) patients in the triple therapy group and 72.7% (32/44) patients in the doublet therapy group have received subsequent treatments post progression. Kaplan-Meier analysis of PPS revealed that patients with post progression treatment (PPTx) displayed significantly improved survival compared with those without in both ICI+TKI+TACE and ICI+TKI groups (**Supplementary Figure S5**).

### Treatment-related adverse events

Treatment-related adverse events (TRAEs) were reported in 40% (24/60) and 45% (27/60) of patients in ICI+TKI+TACE triple therapy group and ICI+TKI doublet therapy group, respectively. The incidence of TRAEs between two groups was not significant (**Supplementary Table S6**). The most frequent TRAEs of any grade in the triple therapy group were rash (n=5, 8.3%) and mucosal inflammation (n=4, 6.6%). In the doublet therapy group, the most frequently reported TRAEs were rash (n=7, 11.6%), diarrhea (n=5, 8.3%), and hypothyroidism (n=5, 8.3%). Grade 3 events occurred in 5 patients (8.3%) in the triple therapy group and in 2 patients (3.3%) in the doublet therapy group. The incidence of TRAEs were summarized in **Supplementary Table S7**. TRAEs were evaluated according to Common Terminology Criteria for version 5.0 (National Cancer Institute) (21).

## Discussion

The success of IMbrave150 trial has launched a new era of ICI-based combination therapies in HCC. So far, the ORR was 29.8% with the combination of atezolizumab plus bevacizumab in the IMbrave 150 study (5), and the highest ORR of systemic therapies for HCC was reported as 36% with pembrolizumab plus lenvatinib in the Keynote 524 study (6). Currently, the efficacy improvement with ICI-based systemic therapies has clearly hit a plateau for HCC. In addition to systemic therapies, LRTs including TACE were also utilized in combination with ICIs to further increase the efficacy (8, 9). Except the conventional role of inducing tumor necrosis, TACE was also found to promote T-cell activation via abscopal effects (22). The tumor necrosis caused by TACE increased the release of tumor-associated antigens (23, 24), which has been proven to recruit DCs and increase AFP-specific CD4^+^T-cell response (25), thus synergizing with ICIs to increase cytotoxic T lymphocytes and decrease tumor-infiltrating Treg cells (26). Lenvatinib was reported to effectively inhibit the angiogenic growth factors triggered by the extensive ischemic necrosis in preclinical (27) or clinical studies (17), which was also an important factor associated with T-cell activation. Recently, several studies have investigated the efficacy of TKI plus ICI in combination with TACE and demonstrated an ORR of 46.7%-69.3% (28–35). The LEAP-012 trial demonstrated a significant and clinically meaningful improvement in PFS for patients with unresectable HCC compared to TACE plus placebo. Similarly, the combination of durvalumab, bevacizumab, and TACE in the EMERALD-1 study also showed potential to establish a new standard of care (36, 37). Nonetheless, the studies comparing the efficacy of ICI+TKI+TACE with ICI+TKI were still limited. Herein, we leveraged a retrospective cohort of HCC patients treated with triple therapy or doublet therapy and firstly revealed that the outcomes were not significantly improved after the addition of TACE to ICI plus TKI combination therapy.

The efficacy of TKI or ICI monotherapy was limited in clinical studies, with an ORR of 6.5% with sorafenib (3), 15-17% with PD-1 inhibitors (4), and 18.8% with lenvatinib (3). The anti-angiogenesis function of TKIs including lenvatinib was involved in several steps of T-cell activation, including the restoration of antigen presentation, the priming and activation of T-cell responses, and the modulation of the tumor immune microenvironment (38, 39). Furthermore, lenvatinib was also found to regulate pathways modulating antitumor immunity, including the reduction of tumor PD-L1 expression levels and Treg differentiation by blocking Fibroblast growth factor receptor-4 (FGFR4) (40) and reducing the Treg proportion via TGF-b pathway inhibition (41). Indeed, the combination therapy of lenvatinib and pembrolizumab initially displayed encouraging efficacy for HCC (6). However, subsequent phase III study LEAP-002 only achieved an ORR of 26.1% and failed to meet its primary endpoint on OS and PFS (7). Similar negative results were also observed in the Cosmic-312 study (42). However, subgroup analysis of LEAP-002 revealed that patients with higher AFP levels, extrahepatic spread or MVI may significantly benefit from lenvatinib plus pembrolizumab, indicating the efficacy of this combination in selected patients (7). Likewise, our data also showed that the outcomes of patients treated with ICI+TKI+TACE triple therapy were not significantly improved versus ICI+TKI doublet therapy. But subgroup and interaction analysis of PFS identified that patients with no preLRT or treated with lenvatinib as combination are more likely to benefit from triple therapy, further highlighting the importance of patient selection in the combination therapy for HCC, which indeed merits further investigation.

LRTs are common regimens to treat patients with uHCC (22). The combinations of LRTs and ICI-based systemic therapies are promising therapeutic strategies and are currently under investigation in HCC (8). Mechanistically, other than tumor elimination, LRT can promote antigenic or immunogenic cell death, thereby augmenting tumor immunogenicity and synergizing with ICIs (8, 43). However, the optimal patient population which is suited for combined locoregional treatments is still not clear. With the recent overwhelming trend of ICI-based combinational therapy in HCC, the triple therapy of ICI+TKI+TACE has increasingly been utilized in clinical practice. However, our real-world data revealed no significant improvement in PFS and OS with triple therapy compared to doublet therapy, suggesting that the addition of TACE to the ICI+TKI combination therapy did not necessarily improve outcomes in the overall population, thereby challenging the notion that “more is better” in HCC combination therapy. Furthermore, the benefit of triple therapy was only observed in selected patients (without preLRT or treated with lenvatinib as combination), emphasizing the importance of tumor heterogeneity and personalized therapy in HCC.

The REFLECT trial and subsequent re-analysis of data showed non-inferiority of lenvatinib versus sorafenib in terms of OS, as well as statistically significant and clinically meaningful improvement in PFS, time to progression, and ORR (3). Lenvatinib is a multitarget tyrosine kinase inhibitor analogous to sorafenib, with unique high selectivity for FGF receptor 1-4 (FGFR1-4) (3). Of the receptors, FGFR4 is considered a potent target of lenvatinib in the treatment of HCC (44), providing a mechanistic rationale for that lenvatinib resulted in a statistically significant improvement in ORR compared with sorafenib in REFLECT trial. Likewise, Yang et al. have reported TACE plus lenvatinib was significantly superior to TACE plus sorafenib with respect to OS, PFS, and ORR (45). In addition, lenvatinib was also found to target FGFR4 to enhance antitumor response of ICI in HCC (40). Consistently, our data showed that patients treated with ICI+lenvatinib displayed superior PFS versus ICI+sorafenib (**Supplementary Table S2**). Importantly, we further revealed that in the patients treated with ICI+lenvatinib, the addition of TACE can remarkably improve PFS in comparison with those treated with ICI+sorafenib, suggesting the synergistic effect between TACE and ICI+lenvatinib.

In the ICI+TKI group, the point of treatment initiation was defined as the initiation of systemic therapy, while in the ICI+TKI+TACE group, it was defined as the initiation of TACE or systemic therapy, whichever comes first. Notably, treatment sequences in landmark trials vary. In LEAP-012 trial, TACE was initiated 2-4 weeks after systemic therapy, while in EMERALD-1 study, durvalumab with or without bevacizumab was administered 7 days post-TACE (36, 37). Moreover, current guidelines also lack consensus on optimal sequencing of TACE and systemic therapy. Due to the nature of real-world studies, the point of treatment initiation was inconsistent between the two groups in our study. Nonetheless, the interval between TACE and systemic therapy was within an acceptable range, and patients in triple therapy group could be considered to have received concurrent TACE and ICI+TKI therapy, and the impact of this inconsistency on the results was largely limited.

In clinical trials, post-progression therapy or treatment crossover after progression increases the fragility of overall survival as an endpoint (46). The COSMIC-312 study (42) only observed an improved PFS in HCC patients treated with cabozantinib plus atezolizumab versus sorafenib but not OS. Similarly, our data also didn’t detect significant interactions between preLRT or TKIs-combined with treatment via interaction analysis of OS, which was probably due to that almost 3/4 progressed patients in both triple therapy and doublet therapy groups had received subsequent post-progression therapies.

This study has several limitations. First, this is a single center retrospective study and almost all patients had an etiology of HBV infection, which limits the generalizability of our findings. Second, the portion of patients with ECOG PS 1 or Child-pugh score B was significantly higher in ICI+TKI doublet therapy group than that in triple therapy group before PS matching (**Supplementary Table S1**), indicating that patients with relatively good performance or preserved liver functions tended to receive ICI+TKI+TACE triple therapy. This bias is probably because, currently, as a potent means of LRT, TACE is preferred to be combined with ICI plus TKI therapy for uHCC when patient conditions permit in the real world. Although PS matching balanced the differences of baseline characteristics between the two groups, our findings still should be confirmed in prospective randomized studies. Third, unlike the LEAP-012 trial (19), in which TACE was limited to 2 treatments per tumor, repeated TACE was allowed in this study, which was also in accordance with the situation in the real world.

## Conclusions

In conclusion, compared with ICI+TKI doublet therapy, ICI+TKI+TACE triple therapy showed minimal difference in the efficacy for uHCC. However, patients with no preLRT or treated with lenvatinib can significantly benefit from ICI+TKI+TACE triple therapy in terms of PFS, providing a rationale for conducting prospective studies to assess the efficacy of adding TACE to ICI plus TKI combination therapy in selected patients with uHCC.

## Data Availability

The raw data supporting the conclusions of this article will be made available by the authors, without undue reservation.
