# Supplementary material for: Immune checkpoint inhibitor plus tyrosine kinase inhibitor with or without transarterial chemoembolization for unresectable hepatocellular carcinoma
Source: Front Oncol. 2025 Mar 10;15:1385304. doi: 10.3389/fonc.2025.1385304 (PMC11930818; doi:10.3389/fonc.2025.1385304)
Supplement: Supplementary file 1 [file DataSheet1.docx]

**Immune Checkpoint Inhibitor plus Tyrosine Kinase Inhibitor with or without Transarterial Chemoembolization for unresectable HCC**

Hongyu Pan^1,#^; Minghao Ruan^1,#^; Riming Jin^1,#^; Jin Zhang^1^; Yao Li^1^; Dong Wu^1^; Lijie Zhang^2,*^; Wen Sun^3,*^; Ruoyu Wang^1,*^.

^1^The First Department of Hepatic Surgery, Eastern Hepatobiliary Surgery Hospital, the Naval Medical University, Shanghai, China.

^2^The Department of Information, Shanghai Changhai Hospital, Naval Medical University, Shanghai, 200438, China.

^3^National Center for Liver Cancer, the Naval Medical University, Shanghai, China.

^#^The authors have contributed equally to this work.

**Running title:**  ICI+TKI+TACE vs. ICI+TKI for uHCC

***Correspondence author and address:**

Dr. Ruoyu Wang (wangruoyu1213@126.com), the First Department of Hepatic Surgery, Eastern Hepatobiliary Surgery Hospital, the Naval Medical University, 225 Changhai Road, 200438 Shanghai, China. Dr. Wen Sun (sunwen_sw@aliyun.com), National Center for Liver Cancer, the Naval Medical University, 366 Qianju Road, Shanghai, 201805, China. Lijie Zhang (jellyzhang.ok@163.com), the Department of Information, Changhai Hospital, the Naval Medical University, 168 Changhai Road, 200438 Shanghai, China.

**Table S1. Baseline characteristics of patients**

|  |  | | **No.(%)** | | | |  | |
| --- | --- | --- | --- | --- | --- | --- | --- | --- |
| **Characteristic** | | **ICI+TKI+TACE**  **group (n=259)** | | **ICI+TKI**  **group (n=81)** | | ***p* value** | |  |
| Age(years) | |  | |  | |  | |  |
|  | ≥60 | | 69 (26.64%) | | 19(23.46%) | | 0.568 | |
|  | <60 | | 190(73.36%) | | 62 (76.54%) | |  | |
| Gender | |  | |  | |  | |  |
|  | Male | | 226(87.26%) | | 69(85.19%) | | <0.001 | |
|  | Female | | 33(12.74%) | | 12(14.81%) | |  | |
| HBV | |  | |  | |  | |  |
|  | Positive | | 245(94.59%) | | 75(92.59%) | | 0.504 | |
|  | Negative | | 14(5.41%) | | 6(7.41%) | |  | |
| HCV | |  | |  | |  | |  |
|  | Positive | 5(1.93%) | | 1(1.23%) | | 0.678 | |  |
|  | Negative | 254(98.07%) | | 80(98.77%) | |  | |  |
| EGOG PS | |  | |  | |  | |  |
|  | 1 | | 77(29.73%) | | 44(54.32%) | | <0.001 | |
|  | 0 | | 182(70.27%) | | 37(45.68%) | |  | |
| Child-Pugh Score | |  | |  | |  | |  |
|  | A | | 240(92.66%) | | 68(83.95%) | | <0.001 | |
|  | B | | 19(7.34%) | | 13(16.05%) | |  | |
| preLRT | | |  | |  | |  | |
|  | Yes | | 107(41.31%) | | 60(74.07%) | | <0.001 | |
|  | No | | 152(58.69%) | | 21(25.93%) | |  | |
| preTKI | |  | |  | |  | |  |
|  | Yes | 27(10.42%) | | 24 (29.63%) | | <0.001 | |  |
|  | No | 232(89.58%) | | 57(70.37%) | |  | |  |
| First-line | |  | |  | |  | |  |
|  | Yes | | 232(89.58%) | | 56(69.14%) | | <0.001 | |
|  | No | | 27(10.42%) | | 25(30.86%) | |  | |
| Previously Untreated | | | | | |  | |  |
|  | Yes | | 133(51.35%) | | 17(20.99%) | | <0.001 | |
|  | No | | 126(48.65%) | | 64 (79.01%) | |  | |
| MVI | | |  | |  | |  | |
|  | present | | 110(42.47%) | | 26(32.10%) | | 0.096 | |
|  | absent | | 149(57.53%) | | 55(67.90%) | |  | |
| Extrahepatic Metastasis | | | | | |  | |  |
|  | present | | 61(23.55%) | | 46 (56.79%) | | <0.001 | |
|  | absent | | 198(76.45%) | | 35(43.21%) | |  | |
| BCLC stage | | |  | |  | |  | |
|  | A | | 31(11.97%) | | 9(11.11%) | | <0.001 | |
|  | B | | 86(33.20%) | | 13(16.05%) | |  | |
|  | C | | 142(54.83%) | | 59(72.84%) | |  | |
| TKIs-combined | | | | | |  | |  |
|  | Sorafenib | | 34(13.13%) | | 26(32.10%) | | <0.001 | |
|  | Lenvatinb | | 225(86.87%) | | 55(67.90%) | |  | |
| TRAEs | | |  | |  | |  | |
|  | Yes | | 88(33.98%) | | 38(46.91%) | | 0.035 | |
|  | No | | 171(66.02%) | | 43(53.09%) | |  | |
| DCP(mAU/mL) | | | |  | |  | |  |
|  | ≥400 | | 164(63.32%) | | 46(56.79%) | | 0.308 | |
|  | <400 | | 90(34.75%) | | 33(40.74%) | |  | |
| AFP(μg/L) | |  | |  | |  | |  |
|  | ≥400 | | 120(46.33%) | | 29(35.80%) | | 0.106 | |
|  | <400 | | 138(53.28%) | | 51(62.96%) | |  | |

Abbreviations: HBV, hepatitis B virus; HCV, hepatitis C virus; TKI, Tyrosine kinase inhibitor; ECOG PS, Eastern Cooperative Oncology Group Performance Status; preLRT, Previous loco-regional therapy; MVI, Macrovascular Invasion; BCLC stage, Barcelona Clinic Liver Cancer stage; AFP, Alpha-fetoprotein; TRAE, Treatment-related adverse events; DCP, Des-gamma-carboxy prothrombin.

**Table S2. Univariable and Multivariable COX Proportional Hazards Regression Model of PFS of patients matched by PSs.**

|  |  | **Univariable Analysis** | | | | |  | **Multivariable Analysis** | | |
| --- | --- | --- | --- | --- | --- | --- | --- | --- | --- | --- |
| **Feature** | | **Median (months)** | | **HR** | **95 %CI** | ***p*** |  | **HR** | **95 %CI** | ***p*** |
| Age(years) | |  | |  |  |  |  |  |  |  |
|  | ≥60 vs <60 | 9.2 vs 7.1 | | 0.77 | 0.48-1.25 | 0.301 |  |  |  |  |
| Gender | |  | |  |  |  |  |  |  |  |
|  | Male vs Female | 7.7 vs 7.5 | | 0.75 | 0.44-1.29 | 0.310 |  |  |  |  |
| HBV | |  | |  |  |  |  |  |  |  |
|  | Positive vs Negative | 7.5 vs 12.9 | | 1.46 | 0.59-3.62 | 0.404 |  |  |  |  |
| HCV | |  | |  |  |  |  |  |  |  |
|  | Positive vs Negative | NR vs 7.5 | | 0.72 | 0.10-5.25 | 0.754 |  |  |  |  |
| ECOG PS | |  | |  |  |  |  |  |  |  |
|  | 1 vs 0 | 9.2 vs 7.1 | 0.72 | | 0.51-1.16 | 0.218 |  |  |  |  |
| Child-Pugh Score | | | | | |  |  |  |  |  |
|  | B vs A | 4.4 vs 8.4 | 2.18 | | 1.21-3.91 | 0.009 |  | 1.84 | 1.01-3.37 | 0.046 |
| preLRT | | | | | | | |  |  |  |
|  | Yes vs No | 8.3 vs 5.7 | 0.90 | | 0.58-1.41 | 0.670 |  |  |  |  |
| preTKI | |  |  | |  |  |  |  |  |  |
|  | Yes vs No | 6.6 vs 7.9 | 1.27 | | 0.79-2.03 | 0.318 |  |  |  |  |
| First-line | |  |  | |  |  |  |  |  |  |
|  | Yes vs No | 7.9 vs 6.6 | 0.78 | | 0.49-1.25 | 0.318 |  |  |  |  |
| Previously Untreated | |  |  | |  |  |  |  |  |  |
|  | Yes vs No | 6.9 vs 8.3 | 1.17 | | 0.73-1.88 | 0.504 |  |  |  |  |
| MVI | | | | | |  |  |  |  |  |
|  | present vs absent | 4.7 vs 9.2 | 1.55 | | 1.01-2.35 | 0.041 |  | 1.26 | 0.82-1.95 | 0.281 |
| Extrahepatic Metastasis | |  |  | |  |  |  |  |  |  |
|  | present vs absent | 10.0 vs 6.7 | 0.69 | | 0.46-1.05 | 0.085 |  |  |  |  |
| BCLC stage | |  |  | |  |  |  |  |  |  |
|  | B vs A | 7.8 vs 6.7 | 1.12 | | 0.58-2.16 | 0.726 |  |  |  |  |
|  | C vs A | 7.7 vs 6.7 | 0.90 | | 0.67-1.21 | 0.519 |  |  |  |  |
| TKIs-combined | | | | | | |  |  |  |  |
|  | Lenvatinb vs Sorafenib | 11.7 vs 4.1 | 0.47 | | 0.30-0.72 | 0.001 |  | 0.53 | 0.34-0.82 | 0.005 |
| DCP(mAU/mL) | | | | | |  |  |  |  |  |
|  | ≥400 vs <400 | 5.7 vs 12.9 | 1.71 | | 1.13-2.57 | 0.010 |  | 1.40 | 0.91-2.16 | 0.115 |
| AFP(μg/L) | |  |  | |  |  |  |  |  |  |
|  | ≥400 vs <400 | 7.0 vs 7.7 | 1.05 | | 0.68-1.61 | 0.818 |  |  |  |  |

Abbreviations: HBV, hepatitis B virus; HCV, hepatitis C virus; TKI, Tyrosine kinase inhibitor; ECOG PS, Eastern Cooperative Oncology Group Performance Status; preLRT, Previous loco-regional therapy; MVI, Macrovascular Invasion; BCLC stage, Barcelona Clinic Liver Cancer stage; AFP, Alpha-fetoprotein; DCP, Des-gamma-carboxy prothrombin.

**Table S3. Univariable and Multivariable COX Proportional Hazards Regression Model of OS of patients matched by PSs**

|  |  | **Univariable Analysis** | | | |  | **Multivariable Analysis** | | |
| --- | --- | --- | --- | --- | --- | --- | --- | --- | --- |
| **Feature** | | **Median (months)** | **HR** | **95 %CI** | **p** |  | **HR** | **95 %CI** | **p** |
| Age(years) | |  |  |  |  |  |  |  |  |
|  | ≥60 vs <60 | 31.9 vs 25.5 | 0.72 | 0.38-1.38 | 0.335 |  |  |  |  |
| Gender | |  |  |  |  |  |  |  |  |
|  | Male vs Female | 26.0vs 22.8 | 0.92 | 0.43-1.96 | 0.836 |  |  |  |  |
| HBV | |  |  |  |  |  |  |  |  |
|  | Positive vs Negative | 26.0 vs 16.9 | 0.93 | 0.33-2.59 | 0.899 |  |  |  |  |
| HCV | |  |  |  |  |  |  |  |  |
|  | Positive vs Negative | NR vs 26.0 | 0.00 |  | 1 |  |  |  |  |
| ECOG PS | |  |  |  |  |  |  |  |  |
|  | 1 vs 0 | 31.9 vs 25.5 | 0.90 | 0.52-1.55 | 0.720 |  |  |  |  |
| Child-Pugh Score | | | | |  |  |  |  |  |
|  | B vs A | 10.0 vs 27.6 | 3.68 | 1.85-7.31 | <0.001 |  | 3.11 | 1.56-6.22 | 0.001 |
| preLRT | | | | | | |  |  |  |
|  | Yes vs No | 31.9 vs 20.0 | 0.61 | 0.34-1.09 | 0.097 |  |  |  |  |
| preTKI | |  |  |  |  |  |  |  |  |
|  | Yes vs No | NR vs 25.3 | 0.62 | 0.30-1.29 | 0.206 |  |  |  |  |
| First-line | |  |  |  |  |  |  |  |  |
|  | Yes vs No | 25.3 vs NR | 1.58 | 0.77-3.25 | 0.206 |  |  |  |  |
| Previously Untreated | |  |  |  |  |  |  |  |  |
|  | Yes vs No | 16.9 vs 27.6 | 1.81 | 0.98-3.32 | 0.055 |  |  |  |  |
| MVI | | | | |  |  |  |  |  |
|  | present vs absent | 15.1 vs 33.2 | 2.85 | 1.64-4.94 | <0.001 |  | 2.54 | 1.46-4.43 | 0.001 |
| Extrahepatic Metastasis | |  |  |  |  |  |  |  |  |
|  | present vs absent | 25.3 vs 26.0 | 1.11 | 0.64-1.92 | 0.689 |  |  |  |  |
| BCLC stage | |  |  |  |  |  |  |  |  |
|  | B vs A | NR vs 25.5 | 0.85 | 0.31-2.36 | 0.769 |  |  |  |  |
|  | C vs A | 25.3 vs 25.5 | 1.15 | 0.74-1.78 | 0.512 |  |  |  |  |
| TKIs-combined | | | | | |  |  |  |  |
|  | Lenvatinb vs Sorafenib | 26.9 vs 19.2 | 0.69 | 0.39-1.22 | 0.211 |  |  |  |  |
| DCP(mAU/mL) | | | | |  |  |  |  |  |
|  | ≥400 vs <400 | 19.2 vs NR | 2.12 | 1.19-3.78 | 0.011 |  | 1.90 | 1.06-3.40 | 0.029 |
| AFP(μg/L) | |  |  |  |  |  |  |  |  |
|  | ≥400 vs <400 | 16.7 vs 27.6 | 1.43 | 0.82-2.50 | 0.196 |  |  |  |  |

Abbreviations: HBV, hepatitis B virus; HCV, hepatitis C virus; TKI, Tyrosine kinase inhibitor; ECOG PS, Eastern Cooperative Oncology Group Performance Status; preLRT, Previous loco-regional therapy; MVI, Macrovascular Invasion; BCLC stage, Barcelona Clinic Liver Cancer stage; AFP, Alpha-fetoprotein; DCP, Des-gamma-carboxy prothrombin.

**Table S4.** **Best Overall Response**

|  | **No.(%)** | |  |
| --- | --- | --- | --- |
|  | **ICI+TKI+TACE**  **(n=60)** | **ICI+TKI**  **(n=60)** | ***p*** |
| CR | 3(5.0%) | 0 (0.0%) |  |
| PR | 7(11.6%) | 13(21.6%) |  |
| SD | 20(33.3%) | 31(51.6%) |  |
| PD | 30(50.0%) | 16(26.6%) |  |
| Objective Response | 10(16.6%) | 13(21.6%) | 0.487 |

Data are presented as n(%). Best overall response is assessed per RECIST version 1.1. CR, Complete response; PR, Partial response; SD, Stable disease; PD, Progressive disease.

**Table S5. Conversion surgery for patients.**

| Conversion | **No.(%)** | |  |
| --- | --- | --- | --- |
|  | **ICI+TKI+TACE** | **ICI+TKI** | ***p*** |
|  | **(n=60)** | **(n=60)** |  |
| Yes | 7(40.0%) | 5(45.0%) | 0.762 |
| No | 53(60.0%) | 55(55.0%) |  |

**Table S6.** **Treatment-related adverse events**

| TRAEs | **No.(%)** | |  |
| --- | --- | --- | --- |
|  | **ICI+TKI+TACE**  **(n=60)** | **ICI+TKI**  **(n=60)** | ***p*** |
| Yes | 24(40.0%) | 27(45.0%) | 0.580 |
| No | 36(60.0%) | 33(55.0%) |  |

TRAEs, Treatment-related adverse events.

**Table S7.** **Details of treatment-related adverse events**

|  | **No.(%)** | | | | | | |
| --- | --- | --- | --- | --- | --- | --- | --- |
| TRAEs | **ICI+TKI+TACE (n=60)** | | |  | **ICI+TKI (n=60)** | | |
|  | **Overall** | **Grade1-2** | **Grade≥3** |  | **Overall** | **Grade1-2** | **Grade≥3** |
| Any TRAEs | 24(40.0%) | 31(51.6%) | 5(8.3%) |  | 27(45.0%) | 39(65.0%) | 2(3.3%) |
| Rash | 5(8.3%) | 5(8.3%) | 0 |  | 7(11.6%) | 6(10.0%) | 1(1.6%) |
| Mucosal inflammation | 4(6.6%) | 3(5.0%) | 1(1.6%) |  | 3(5.0%) | 3(5.0%) | 0 |
| Diarrhoea | 3(5.0%) | 3(5.0%) | 0 |  | 5(8.3%) | 5(8.3%) | 0 |
| Pruritus | 3(5.0%) | 3(5.0%) | 0 |  | 1(1.6%) | 1(1.6%) | 0 |
| Decreased appetite | 3(5.0%) | 3(5.0%) | 0 |  | 0 | 0 | 0 |
| Hypertension | 3(5.0%) | 3(5.0%) | 0 |  | 2(3.3%) | 2(3.3%) | 0 |
| Asthenia | 3(5.0%) | 3(5.0%) | 0 |  | 0 | 0 | 0 |
| Hypothyroidism | 2(3.3%) | 2(3.3%) | 0 |  | 5(8.3%) | 5(8.3%) | 0 |
| Pyrexia | 2(3.3%) | 2(3.3%) | 0 |  | 2(3.3%) | 2(3.3%) | 0 |
| Thrombocytopenia | 2(3.3%) | 2(3.3%) | 0 |  | 3(5.0%) | 2(3.3%) | 1(1.6%) |
| Interstitial pneumonia | 2(3.3%) | 2(3.3%) | 0 |  | 0 | 0 | 0 |
| Transaminitis | 2(3.3%) | 0 | 2(3.3%) |  | 1(1.6%) | 1(1.6%) | 0 |
| Arthralgia | 1(1.6%) | 1(1.6%) | 0 |  | 2(3.3%) | 2(3.3%) | 0 |
| Heart failure | 1(1.6%) | 1(1.6%) | 0 |  | 1(1.6%) | 1(1.6%) | 0 |
| Hand-foot syndrome | 1(1.6%) | 1(1.6%) | 0 |  | 1(1.6%) | 1(1.6%) | 0 |
| Abdominal pain | 0 | 0 | 0 |  | 2(3.3%) | 2(3.3%) | 0 |
| Hepatitis | 1(1.6%) | 0 | 1(1.6%) |  | 0 | 0 | 0 |
| Hypoalbuminaemia | 1(1.6%) | 0 | 1(1.6%) |  | 0 | 0 | 0 |
| Alopecia | 1(1.6%) | 1(1.6%) | 0 |  | 0 | 0 | 0 |
| Hypotension | 1(1.6%) | 1(1.6%) | 0 |  | 0 | 0 | 0 |
| Hoarseness | 0 | 0 | 0 |  | 1(1.6%) | 1(1.6%) | 0 |
| Weight Decrease | 0 | 0 | 0 |  | 1(1.6%) | 1(1.6%) | 0 |
| Throat irritation | 0 | 0 | 0 |  | 1(1.6%) | 1(1.6%) | 0 |
| Leukopenia | 0 | 0 | 0 |  | 0 | 0 | 0 |
| Hypoadrenalism | 0 | 0 | 0 |  | 1(1.6%) | 1(1.6%) | 0 |
| Hypersensitivity | 0 | 0 | 0 |  | 1(1.6%) | 1(1.6%) | 0 |
| Osteoporosis | 0 | 0 | 0 |  | 1(1.6%) | 1(1.6%) | 0 |

TRAEs, Treatment-related adverse events.


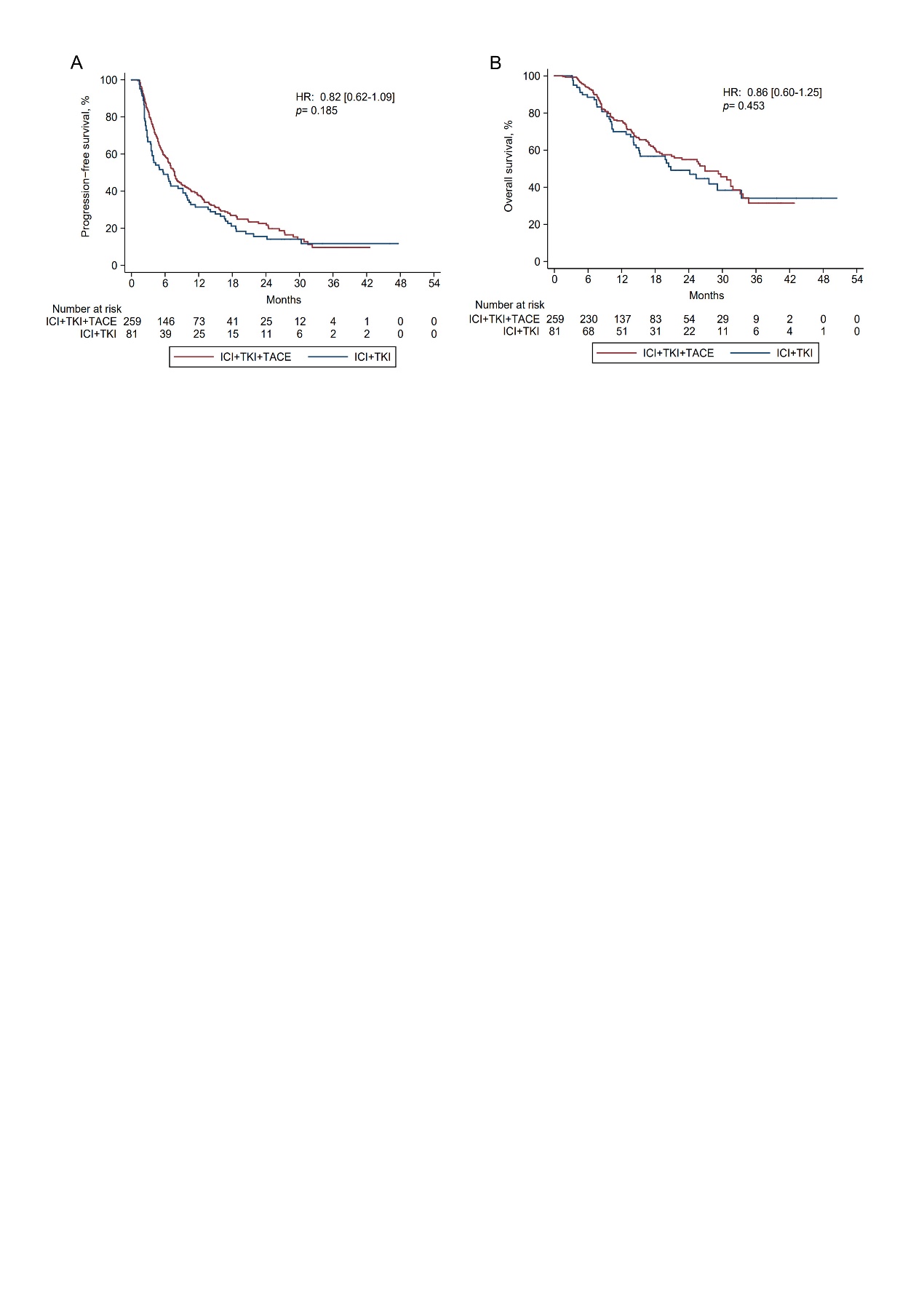


**Figure S1.** Kaplan-Meier estimates of PFS (A) and OS (B) curves in HCC patients treated with ICI+TKI+TACE or ICI+TKI therapy prior to PSM.


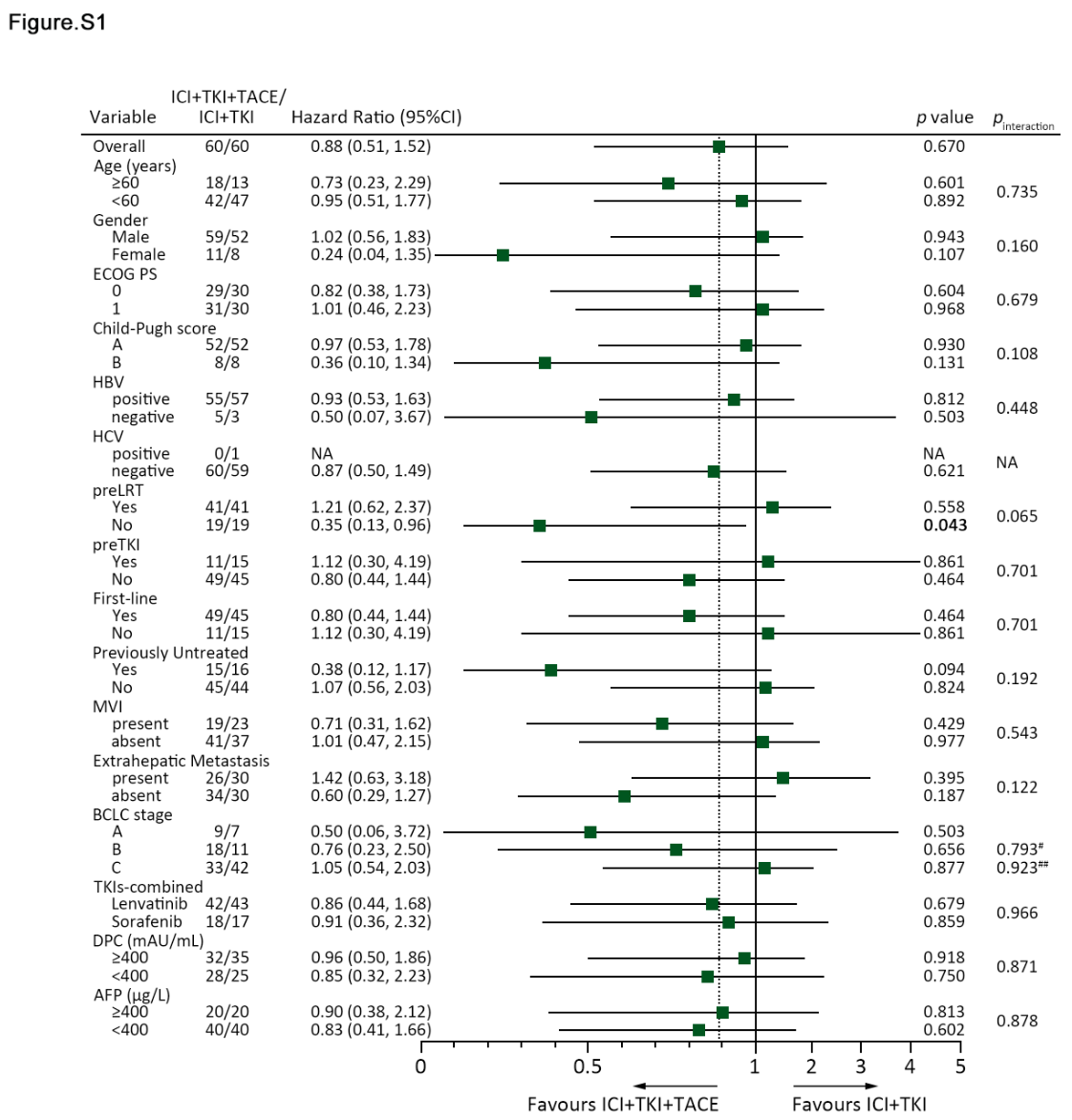


**Figure.S2** Subgroup COX proportional hazards regression model analysis of OS according to the baseline characteristics and different treatment groups. ^#^, BCLC stage B versus A; ^##^, BCLC stage C versus A.

**
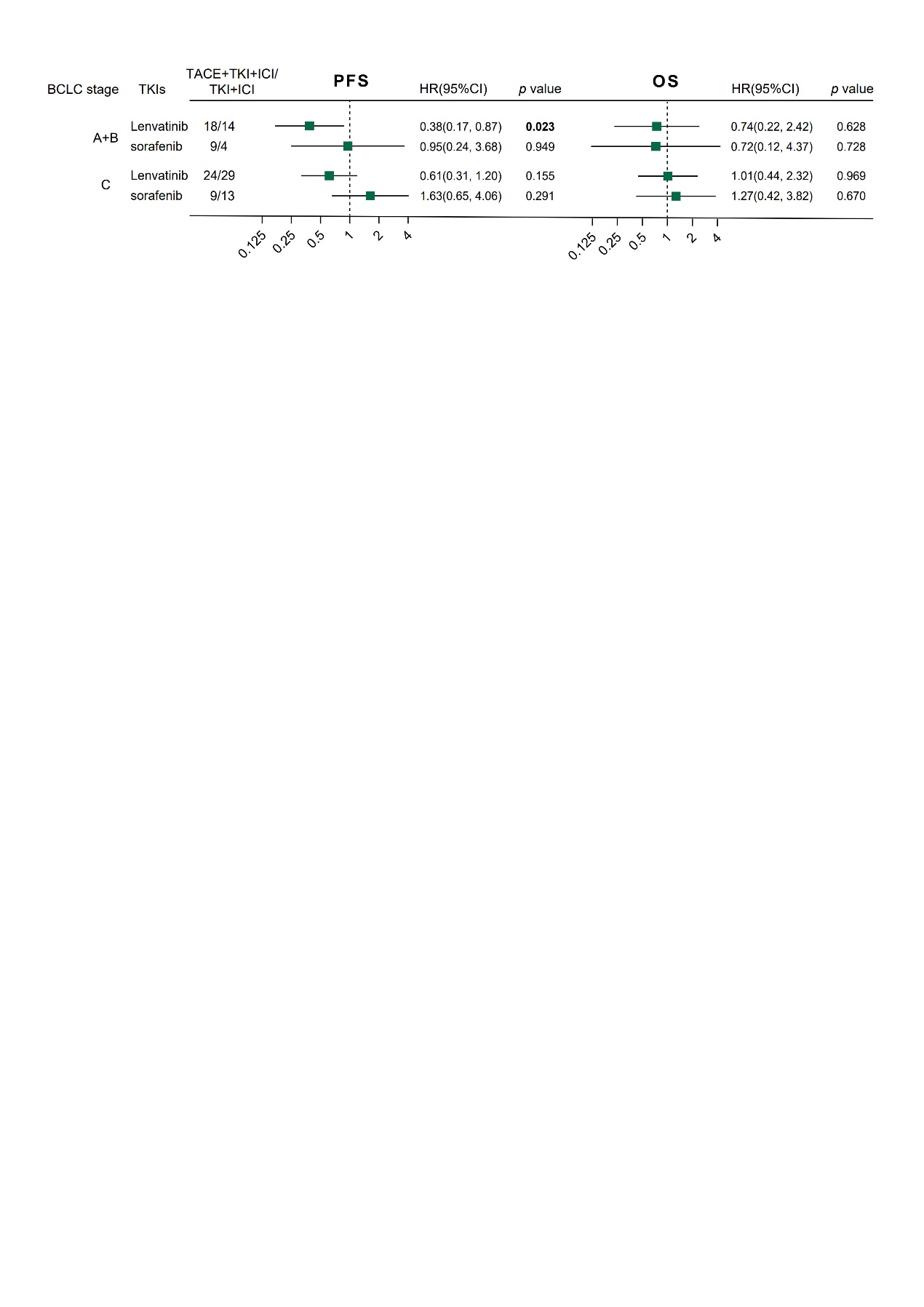
Figure.S3** Forest plot for subgroup COX proportional hazards regression model analysis of PFS and OS according to BCLC stage and different treatment groups.


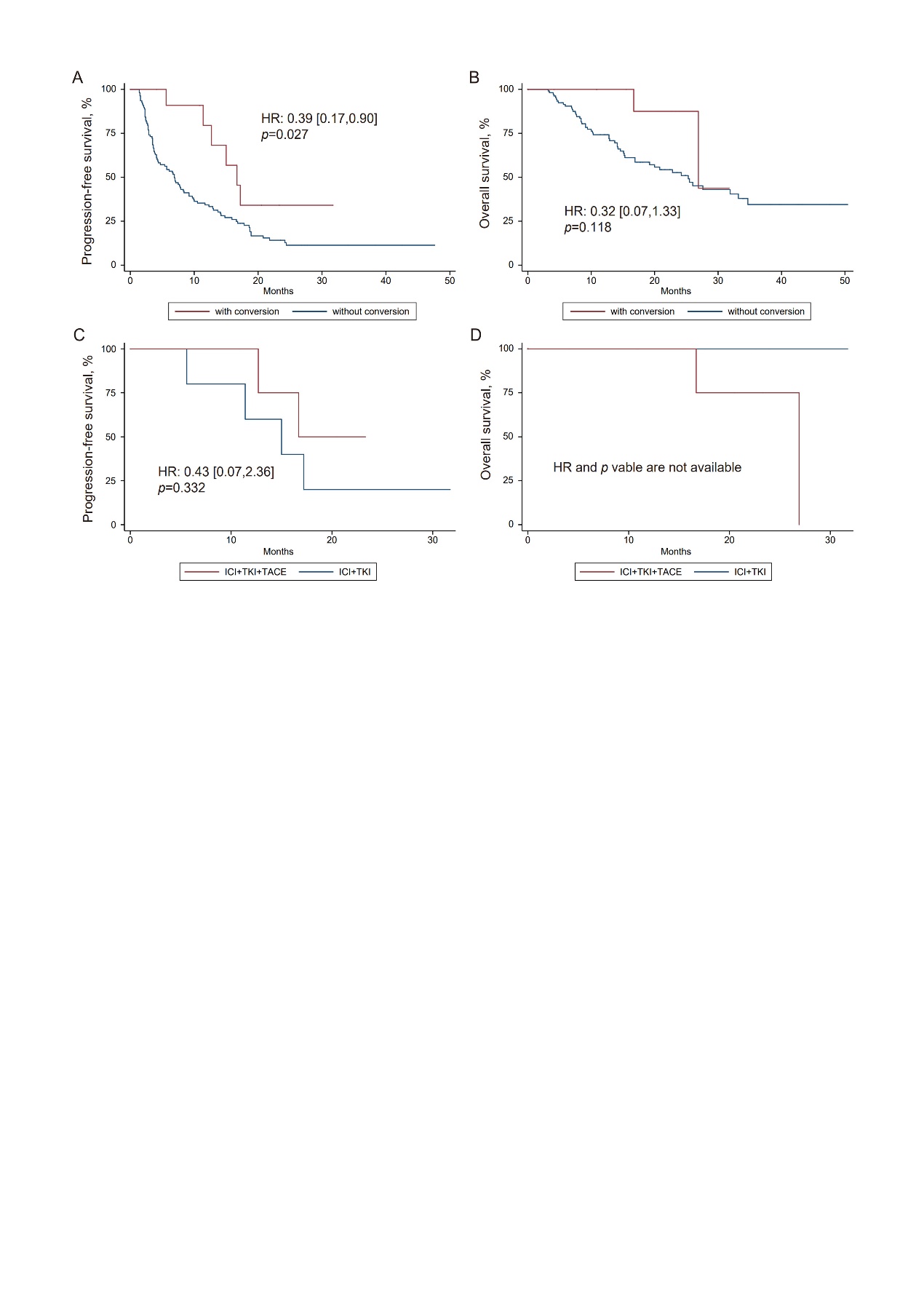


**Figure.S4** (A&B) The PFS and OS were compared between patients treated with and without subsequent conversion surgery. (C&D) The PFS and OS were compared between the triple and doublet therapy groups in patients treated with subsequent conversion surgery.


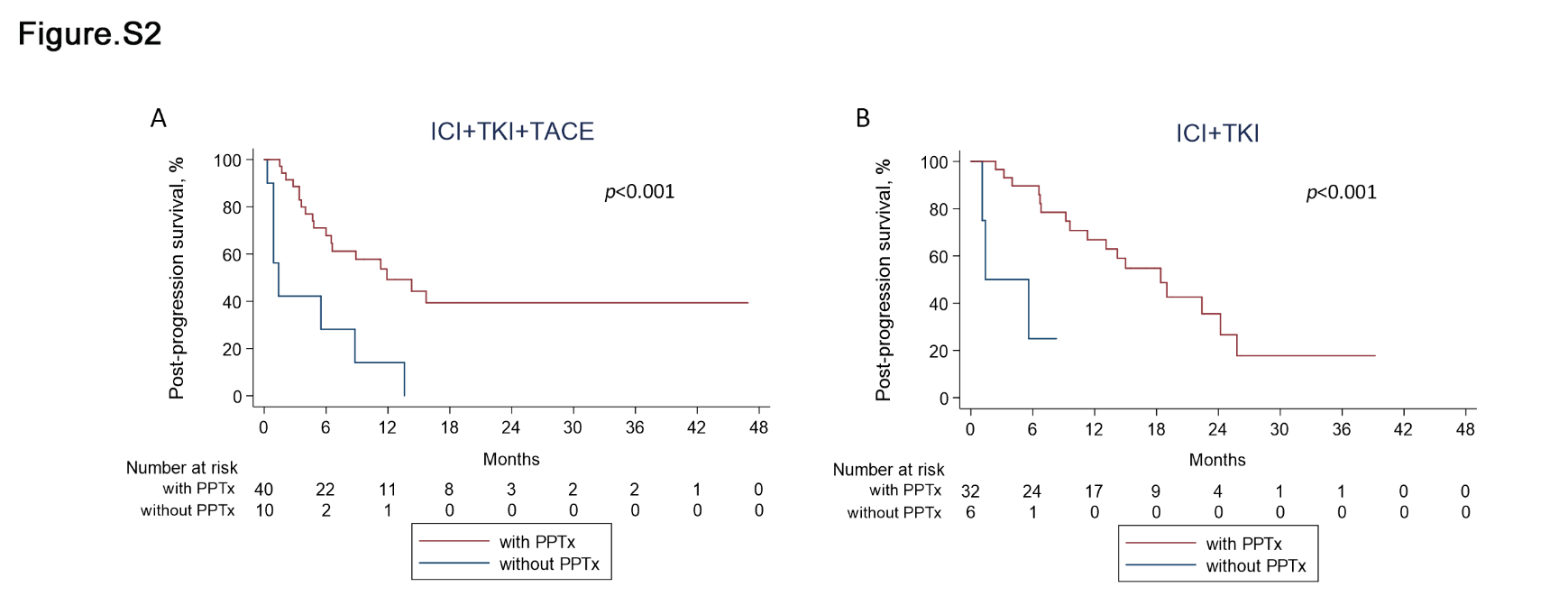


**Figure.S5** Kaplan-Meier estimates of PPS curves in HCC patients treated with ICI+TKI+TACE (A) or ICI+TKI (B) therapy stratified by post progression treatment (PPTx).
